# Polyfluoroalkyl Compounds in Texas Children from Birth through 12 Years of Age

**DOI:** 10.1289/ehp.1104325

**Published:** 2011-12-19

**Authors:** Arnold Schecter, Noor Malik-Bass, Antonia M. Calafat, Kayoko Kato, Justin A. Colacino, Tyra L. Gent, Linda S. Hynan, T. Robert Harris, Sunitha Malla, Linda Birnbaum

**Affiliations:** 1 University of Texas School of Public Health, Dallas, Texas, USA; 2 Division of Laboratory Sciences, National Center for Environmental Health, Centers for Disease Control and Prevention, Atlanta, Georgia, USA; 3 Department of Environmental Health Sciences, University of Michigan School of Public Health, Ann Arbor, Michigan, USA; 4 Department of Clinical Sciences (Biostatistics), and; 5 Department of Psychiatry, University of Texas Southwestern Medical Center, Dallas, Texas, USA; 6 National Institute of Environmental Health Sciences, National Institutes of Health, Department of Health and Human Services, Research Triangle Park, North Carolina, USA

**Keywords:** blood, children, infants, PFC, PFOA, PFOS, polyfluoroalkyl compounds, United States

## Abstract

Background: For > 50 years, polyfluoroalkyl compounds (PFCs) have been used worldwide, mainly as surfactants and emulsifiers, and human exposure to some PFCs is widespread.

Objectives: Our goal was to report PFC serum concentrations from a convenience sample of Dallas, Texas, children from birth to < 13 years of age, and to examine age and sex differences in PFC concentrations.

Methods: We analyzed 300 serum samples collected in 2009 for eight PFCs by online solid phase extraction–high performance liquid chromatography–isotope dilution–tandem mass spectrometry.

Results: Perfluorohexane sulfonic acid (PFHxS), perfluorooctane sulfonic acid (PFOS), perfluorooctanoic acid (PFOA), and perfluorononanoic acid (PFNA) were detected in > 92% of participants; the other PFCs measured were detected less frequently. Overall median concentrations of PFOS (4.1 ng/mL) were higher than those for PFOA (2.85 ng/mL), PFNA (1.2 ng/mL), and PFHxS (1.2 ng/mL). For PFOS, PFOA, PFNA, and PFHxS, we found no significant differences (*p* < 0.05) by sex, significantly increasing concentrations for all four chemicals by age, and significantly positive correlations between all four compounds.

Conclusions: We found no significant differences in the serum concentrations of PFOS, PFOA, PFNA, and PFHxS by sex, but increasing concentrations with age. Our results suggest that these 300 Texas children from birth through 12 years of age continued to be exposed to several PFCs in late 2009, years after changes in production of some PFCs in the United States.

Polyfluoroalkyl compounds (PFCs) are a group of organic chemicals that contain a fluorinated hydrophobic carbon chain attached to hydrophilic heads. Certain PFCs are persistent and nonbiodegradable and can be transported over long distances in the environment (Egeghy and Lorber 2010). Because of these properties, some PFCs have been included in the Stockholm Convention on Persistent Organic Pollutants (POPs) (Stockholm Convention 2008). Like other POPs, some PFCs have a long serum half-life in humans ranging from 3.5 to 7.3 years (Olsen et al. 2007). PFCs include perfluorooctane sulfonic acid (PFOS) and its precursor perfluorooctane sulfonamide (PFOSA), perfluorooctanoic acid (PFOA), perfluorononanoic acid (PFNA), and perfluorohexane sulfonic acid (PFHxS) (Fromme et al. 2009). The most frequently detected PFCs in humans are PFOS and PFOA (Fromme et al. 2009).

For the last 50 years, PFCs have been used worldwide in various settings (Kannan et al. 2004; Kato et al. 2009a; Lindstrom et al. 2011; Schecter et al. 2010), primarily as surfactants and emulsifiers. Some examples include their use in food packaging, nonstick coatings, firefighting foams, paper coatings, and textile coatings (especially carpet and upholstery) and in personal care products such as cosmetics, lotions, nail polish, and shaving cream (Ohio Department of Health 2006). Because of recent findings of the toxicity of these compounds, efforts are being made to reduce or eliminate their use (Lindstrom et al. 2011). In 2002, for example, 3M, a major North American PFC producer, ended production of PFOS-related compounds, the ammonium salt of PFOA, and other perfluorooctanesulfonyl fluoride–based products (3M Company 2000; Prevedouros et al. 2005). Studies conducted after 2002 have shown PFOA and PFOS serum concentrations in adults to be lower than reported before 2002, whereas PFNA concentrations appear to be increasing (Calafat et al. 2007b; Haug et al. 2009; Kato et al. 2011). Therefore, although steps are being taken that may decrease the presence of PFCs, these toxic compounds continue to persist in our environment (Lindstrom et al. 2011).

Human exposure to PFCs can occur through indoor and outdoor air inhalation, dust inhalation ingestion, and ingestion of contaminated food or water (Fromme et al. 2009). Major exposure pathways for children may also include dust and soil ingestion, placental transfer, and breast-feeding (Fromme et al. 2009; Hanssen et al. 2010; Kärrman et al. 2007; Monroy et al. 2008; Zhang et al. 2010a). PFCs have been detected in infant, child, and adult blood or serum samples and in adult liver and breast milk (Frisbee et al. 2009; Kärrman et al. 2010; Liu et al. 2010; Llorca et al. 2010; Tao et al. 2008a, 2008b; Zhang et al. 2010b). Serum concentrations of some PFCs, such as PFOA, have been associated with age, sex, and race (Calafat et al. 2007a, 2007b; Steenland et al. 2009).

Some studies have examined associations between PFC exposures in children and health effects, with inconsistent results (Lindstrom et al. 2011; Steenland et al. 2009). A recent study by Gump et al. (2011) reported an association between PFC levels and impaired response inhibition in children. A cross-sectional study measuring sexual maturation in a 2005–2006 survey of 3,076 Mid-Ohio Valley boys and 2,931 Mid-Ohio Valley girls 8–18 years of age who were exposed to drinking water contaminated with PFOA reported that PFOA and PFOS serum concentrations were associated with later sexual maturation in boys and girls (Lopez-Espinosa et al. 2011). In contrast, a study of 218 girls reporting menarche before the age of 11.5 years, and 230 girls reporting menarche after the age of 11.5 years from the general U.K. population born between 1 April 1991 and 31 December 1992 showed no association between prenatal PFC exposure and age at menarche (Christensen et al. 2011). An American study by Pinney et al. (2009) reported that serum PFOA concentrations were positively correlated with earlier breast maturation and inversely correlated with body mass index in 689 girls 6–7 years of age.

Assessing human exposure to PFCs can provide useful information for understanding their potential adverse health effects. However, such exposure data are rather limited for infants and young children. In the United States, the Centers for Disease Control and Prevention (CDC), as part of the National Health and Nutrition Examination Survey (NHANES), provides information on PFC exposures among older children based on serum concentrations in a representative sample of the U.S. general population ≥ 12 years of age (Kato et al. 2011). For younger children within the general U.S. population, data on the concentrations of PFCs are available from 598 children, 2–12 years of age, recruited from a multicenter clinical trial in 1994–1995 (Olsen et al. 2004) and from pooled sera collected from 936 children 3–11 years of age who were NHANES participants in 2001–2002 (Kato et al. 2009a). More recently, as part of the C8 Health Study, serum concentrations of PFCs were measured in study area residents, including children 1–13 years of age (Frisbee et al. 2009, 2010). The C8 Health Project was created and funded by a settlement that resulted from PFOA-contaminated drinking water and included > 60,000 participants. However, levels in this study population are not likely to be representative of U.S. children as a whole because of relatively high environmental exposures.

For this study, we measured serum concentrations of eight PFCs in a convenience sample of 300 infants and children from birth through 12 years of age who had blood samples collected at a children’s medical center in Dallas, Texas, in 2009.

## Methods

This study was reviewed and found to be exempt from informed consent requirements by the institutional review board committees for the protection of human subjects at the University of Texas Southwestern Medical Center, the University of Texas Health Science Center at Houston, and the CDC.

*Sample collection.* Serum samples were collected from August 2009 through November 2009 at Children’s Medical Center in Dallas, Texas, from a convenience sample of 300 children < 13 years of age undergoing blood tests. It is unknown if serum was collected from healthy children or if these children were receiving evaluation or treatment for illness. Samples were stored frozen at –80°C after collection, and approximately 0.5–1.0 mL residual serum from each child was sent on dry ice to CDC for chemical analysis. No demographic or anthropometric information was available other than the child’s date of birth (DOB), date of serum collection, age (calculated based on DOB and date of serum collection), and sex.

*Chemical analyses.* Serum concentrations of eight PFCs—PFOSA, 2-(*N*-ethyl-perfluorooctane sulfonamido) acetic acid (Et-PFOSA-AcOH), 2-(*N*-methyl-perfluorooctane sulfonamido) acetic acid (Me-PFOSA-AcOH), PFHxS, PFOS, PFOA, PFNA, and perfluorodecanoic acid (PFDA)—were measured by online solid phase extraction–high performance liquid chromatography–isotope dilution–tandem mass spectrometry as previously described (Kato et al. 2011). The limits of detection (LOD) in 0.1 mL serum were 0.1 ng/mL (PFOA, PFNA, PFHxS, PFOSA) and 0.2 ng/mL (PFOS, PFDA, Et-PFOSA-AcOH, Me-PFOSA-AcOH).

*Statistical analyses.* Concentrations below the LOD were assigned a value equal to the LOD divided by the square root of 2 for statistical analyses. Statistical analyses were done only on chemicals with > 92% detection rates, except for a comparison of Me-PFOSA-AcOH concentrations in children ≤ 4 years of age versus > 4 years of age. We calculated univariate statistics, including medians, ranges, and detection frequencies, for each PFC. Analyses were conducted separately for boys and girls and for boys and girls combined. Because PFC concentrations were highly skewed, we used the Wilcoxon rank-sum test to compare PFC concentrations between boys and girls. The Kruskal–Wallis test, a nonparametric analysis of variance, was used to examine overall differences in median concentrations among age groups (0 to < 3 years, 3 to < 6 years, 6 to < 9 years, and 9 to < 13 years) for girls, boys, and girls and boys combined. When the Kruskal–Wallis test was significant, we used Dunn’s method to perform a nonparametric test of differences among multiple groups with unequal sample sizes and tied ranks (Elliott and Hynan 2011). To assess associations between age and individual PFC serum concentrations, we calculated Spearman rank-order correlations. IBM^©^ SPSS Statistics version 19 (SPSS, Inc., IBM, Chicago, IL,) and SAS version 9.2 (SAS Institute Inc., Cary, NC) were used to analyze these data; significance was set to *p* < 0.05 unless otherwise noted.

## Results

We measured eight PFCs, including the four PFCs most frequently detected in NHANES, in 300 serum samples collected in 2009 from Texas children (157 girls and 143 boys) ranging from birth to < 13 years of age (Table 1). Frequencies of detection of the analytes ranged from < 1% to 98% and were > 92% for PFHxS, PFOS, PFOA, and PFNA (Table 2). PFOSA and Et-PFOSA-AcOH were detected in < 2% of samples; Me-PFOSA-AcOH was detected in 36%, and PFDA was detected in 14% of the samples. For girls and boys combined, median serum concentrations were 1.20 ng/mL for PFHxS and PFNA, 2.85 ng/mL for PFOA, and 4.10 ng/mL for PFOS (Table 2). Infants < 1 year of age (*n* = 25) had PFHxS concentrations ranging from nondetectable to 4.0 ng/mL (median, 0.5 ng/mL), PFNA concentrations from nondetectable to 2.2 ng/mL (median, 0.6 ng/mL), PFOA from nondetectable to 8.8 ng/mL (median, 1.9 ng/mL), and PFOS from nondetectable to 6 ng/mL (median, 2.4 ng/mL).

**Table 1:** Age and sex distribution (*n*) of 300 children from Dallas, Texas.

Age (years)	Boys	Girls	All
0 to < 1		15		10		25
1 to < 2		13		12		25
2 to < 3		16		9		25
3 to < 4		18		7		25
4 to < 5		10		15		25
5 to < 6		13		12		25
6 to < 7		12		13		25
7 to < 8		9		16		25
8 to < 9		7		18		25
9 to < 10		11		14		25
10 to < 11		11		13		24
11 to < 12		6		8		14
12 to < 13		2		10		12
Total		143		157		300


**Table 2:** Summary statistics for serum concentrations in 300 children 0–12 years of age from Dallas, Texas, 2009.

Detection frequency (%)					LOD (ng/mL)	Median (ng/mL)					Maximum (ng/mL)		
Boys (*n* = 143)	Girls (*n* = 157)												
		PFC	All	Boys		Girls	All	Boys	Girls
PFOA		97		99		98		0.1		2.70		2.90		2.85		13.50		9.60
PFOS		94		97		96		0.2		4.20		4.10		4.10		93.30		38.30
PFNA		95		99		97		0.1		1.20		1.30		1.20		55.80		4.20
PFHxS		93		92		93		0.1		1.20		1.10		1.20		31.20		9.60
Et-PFOSA-AcOH		0		< 1		< 1		0.2		< 0.2		< 0.2		< 0.2		< 0.2		0.70
Me-PFOSA-AcOH		36		36		36		0.2		< 0.2		< 0.2		< 0.2		28.90		13.40
PFDA		13		15		14		0.2		< 0.2		< 0.2		< 0.2		1.00		2.10
PFOSA		1		1		1		0.1		< 0.1		< 0.1		< 0.1		0.60		0.30


Median concentrations were very similar for girls and boys (Table 2). Further, we did not observe statistically significant differences in serum concentrations by sex for any of the PFCs examined (all Wilcoxon rank-sum tests *p* > 0.05).

For each of the four PFCs with detection frequencies > 92% (PFHxS, PFOS, PFOA, and PFNA), the Kruskal–Wallis test of overall differences among age groups (0 to < 3, 3 to < 6, 6 to < 9, 9 to < 13) was significant for boys alone, girls alone, and boys and girls combined (Table 3). For all children combined and girls only, median values for all four PFCs were significantly lower for children 0 to < 3 years of age compared with the older age groups. For boys, median concentrations were not significantly different between 3 to < 6 years and 0 to < 3 years age groups, but there were some significant differences for older age groups. Median values increased monotonically by age group for PFOS and PFHxS (in girls, boys, and girls and boys combined) but were generally consistent by age > 0 to < 3 years for PFOA and PFNA.

**Table 3:** Summary statistics by age group for serum concentrations of PFOS, PFOA, PFNA, and PFHxS in children 0 to < 13 years of age from Dallas, Texas.

Age group (years)																							Kruskal–Wallis *p-*Value	Post hoc pairwise testing (Dunn method) *p*-values								
0 to < 3					3 to < 6					6 to < 9					9 to < 13					0 to < 3 vs.					3 to < 6 vs.		
PFC/Sex		*n*	Med	Max	*n*	Med	Max	*n*	Med	Max	*n*	Med	Max	3 to < 6	6 to < 9	9 to < 13	6 to < 9	9 to < 13					
PFOS ng/mL																																					
	Boys		44		2.3		10.5		41		2.8		23.3		28		6		76		30		7.05		93.3		< 0.0001				< 0.001		< 0.001		0.020		0.011
	Girls		31		1.8		10.6		34		4.25		14.4		47		4.3		21.7		45		5.6		38.3		< 0.0001		< 0.001		< 0.001		< 0.001				
	Combined		75		2		10.6		75		3.7		23.3		75		5		76		75		6.3		93.3		< 0.0001		< 0.01		< 0.001		< 0.001				< 0.01
PFOA ng/mL																																					
	Boys		44		2.2		8.8		41		2.6		11.1		28		3.75		10.7		30		3.45		13.5		0.021				0.036						
	Girls		31		1.8		9.6		34		3.3		6.2		47		2.9		7.6		45		3		6.1		0.0001		< 0.001		< 0.01		< 0.01				
	Combined		75		2		9.6		75		3.1		11.1		75		3		10.7		75		3		13.5		< 0.0001		< 0.001		< 0.001		< 0.001				
PFNA ng/mL																																					
	Boys		44		0.9		55.8		41		1.1		9.2		28		1.4		14.4		30		1.3		6		0.0091				0.013						
	Girls		31		0.6		2.2		34		1.45		4.2		47		1.4		3.9		45		1.4		3.3		< 0.0001		< 0.001		< 0.001		< 0.001				
	Combined		75		0.6		55.8		75		1.3		9.2		75		1.4		14.4		75		1.3		6		< 0.0001		< 0.001		< 0.001		< 0.001				
PFHxS ng/mL																																					
	Boys		44		0.6		4		41		1		14.6		28		1.5		31.2		30		1.7		9		0.0004				< 0.01		< 0.01				
	Girls		31		0.5		2		34		1.2		3.3		47		1.3		9.6		45		1.6		5.4		< 0.0001		< 0.001		< 0.001		< 0.001				
	Combined		75		0.5		4		75		1.1		14.6		75		1.3		31.2		75		1.6		9		< 0.0001		< 0.001		< 0.001		< 0.001				
Abbreviations: Med, median; Max, maximum.																																					

Serum concentrations of PFOS, PFNA, PFHxS, and PFOA were significantly correlated with age (Spearman rank correlations 0.168–0.425, all *p* < 0.04) (Table 4). Correlations between PFOS, PFOA, PFNA, and PFHxS concentrations were positive and significant among girls, boys, and girls and boys combined (Spearman rank correlations 0.525–0.812, all *p* < 0.0001).

**Table 4:** Spearman rank order correlations (rho) for age and serum concentrations of PFOS, PFOA, PFNA, and PFHxS.

Age at collection			PFOS			PFOA			PFNA			PFHxS		
Group/PFC		Spearman rho	*p*-Value	Spearman rho	*p*-Value	Spearman rho	*p*-Value	Spearman rho	*p*-Value	Spearman rho	*p*-Value
Boys (*n* = 143)																					
	PFOS		0.425		< 0.0001						0.805		< 0.0001		0.737		< 0.0001		0.812		< 0.0001
	PFOA		0.264		0.0014		0.805		< 0.0001						0.770		< 0.0001		0.811		< 0.0001
	PFNA		0.273		0.0010		0.737		< 0.0001		0.770		< 0.0001						0.648		< 0.0001
	PFHxS		0.376		< 0.0001		0.812		< 0.0001		0.811		< 0.0001		0.648		< 0.0001				
Girls (*n* = 157)																					
	PFOS		0.402		< 0.0001						0.726		< 0.0001		0.632		< 0.0001		0.764		< 0.0001
	PFOA		0.168		0.0356		0.726		< 0.0001						0.763		0.0000		0.635		< 0.0001
	PFNA		0.215		0.0069		0.632		< 0.0001		0.763		< 0.0001						0.525		< 0.0001
	PFHxS		0.423		< 0.0001		0.764		< 0.0001		0.635		< 0.0001		0.525		< 0.0001				
Combined (boys and girls *n* = 300																					
	PFOS		0.421		< 0.0001						0.768		< 0.0001		0.684		< 0.0001		0.784		< 0.0001
	PFOA		0.220		0.0001		0.768		< 0.0001						0.766		< 0.0001		0.730		< 0.0001
	PFNA		0.251		< 0.0001		0.684		< 0.0001		0.766		< 0.0001						0.583		< 0.0001
	PFHxS		0.397		< 0.0001		0.784		< 0.0001		0.730		< 0.0001		0.583		< 0.0001				


## Discussion

Our results suggest that serum concentrations of PFOA, PFOS, PFNA, and PFHxS in our study population increased from birth until < 13 years of age. Those in the youngest group, 0 to < 3 years, have significantly lower PFC serum concentrations than those in groups 6 to < 9 or 9 to < 13, except for PFOA and PFNA in boys. It is possible that concentrations may increase with age partly because of cumulative exposures to PFCs via food and dust intake (Fromme et al. 2009). It is also possible that younger children in our study population experienced lower exposure to some PFCs than older children, because PFOS production stopped in 2002 in the United States (3M Company 2000), and PFOA release during manufacturing has declined since the early 2000s (U.S. Environmental Protection Agency 2006). The discontinuation of production of some of these chemicals, coupled with their relatively long half-life, could explain why older children had higher levels of these chemicals. However, the relevance of the changes in manufacturing practices is difficult to determine, given the persistent nature of PFCs and the lack of information on baseline levels (e.g., PFC concentrations in the early 2000s for the older children) and on exposure pathways for our study population. Except for age and sex of the children, information on individual factors that might influence PFC levels, such as breast-feeding history, was not available, thus making it difficult to assess potential determinants of exposure (Fromme et al. 2010).

Others have reported an association between age and PFC serum concentrations. Zhang et al. (2010b) observed significant positive associations between age and whole blood concentrations of several PFCs, including PFHxS, PFOS, PFNA, and PFDA, but not PFOA, in 184 Chinese children 0–10 years of age. The authors speculated that these associations might have been related to dietary exposures, as PFCs have been detected in food worldwide (Zhang et al. 2010b). A positive association between age and PFC serum concentrations was also reported among adults in Norway and Australia (Haug et al. 2009; Kärrman et al. 2006) but was not evident in a study of convenience samples of blood, plasma, and serum from volunteer donors in the United States, Colombia, Belgium, Malaysia, Korea, and other countries (20–75 samples per source population) (Kannan et al. 2004).

There are conflicting data regarding sex differences in PFC concentrations in children. Zhang et al. (2010b) noted that median concentrations of four PFCs, including PFNA, PFOA, and PFOS, were slightly higher in whole blood samples from Chinese females (*n* = 82) compared with males (*n* = 163) 0–90 years of age. Results from a study using sera collected in southeast Queensland, Australia, in 2006–2007 from 2,420 donors 0 to > 60 years of age and pooled according to age showed no apparent sex differences in children (< 12 years of age) (Toms et al. 2009). In the present study, we also did not find a difference in children’s PFC serum concentrations based on sex.

The higher detection frequency for Me-PFOSA-AcOH versus Et-PFOSA-AcOH in our study population may be related to the use of these chemicals and subsequent exposure routes for children: Me-PFOSA-AcOH is an oxidation product of 2-(*N*-methyl-perfluorooctane sulfonamide) ethanol, used in carpets and textiles, whereas Et-PFOSA-AcOH is used in paper products (Kato et al. 2009a; Olsen et al. 2003).

In this study, we report serum PFC concentrations, which are measures of internal exposure, without attempting to address external exposures in a population of children in Dallas, Texas. These values were lower than concentrations measured previously by others at different times, locations, and ages both in the United States and other countries. Differences in PFC concentrations in our study population compared with others may also be related to age, country, use of whole blood versus serum, pooled versus individual sampling, or the timing of specimen collection, which occurred later than in other studies and several years after production of some PFCs ceased in the United States (Calafat et al. 2006, 2007a, 2007b; CDC 2010; Guruge et al. 2005; Hemat et al. 2010; Hölzer et al. 2008; Kato et al. 2009b; Olsen et al. 2004; Spliethoff et al. 2008; Toms et al. 2009; Zhang et al. 2010b).

Higher serum concentrations of PFOS, PFHxS, and PFOA were reported by Frisbee et al. (2009, 2010) for children living in areas of the United States with known environmental PFOA contamination. However, PFNA concentrations were similar in the C8 study population (median, 1.6 ng/ mL) and our study population (median, 1.2 ng/ mL) (Frisbee et al. 2009, 2010). Median concentrations from birth to < 13 years were lower in our study population than median concentrations reported for NHANES 2007–2008 ages 12–19 years (357 samples) for PFOS (11.3 ng/mL), PFOA (4.0 ng/mL), and PFHxS (2.3 ng/mL) and slightly higher for PFNA (1.4 ng/mL) (Kato et al. 2011). Our findings for Texas children from birth through 1 year in 2009 are consistent with ranges of median values reported for 110 pooled blood spot samples taken at birth for congenital disease testing in 2,640 New York infants born between 1997 and 2007 (Spliethoff et al. 2008). Concentrations in children from birth through age 1 year are also consistent with median concentrations in dried blood spots (generally from 2-day-old children) from 98 infants born in Texas in May 2007 (Kato et al. 2009b).

Dried blood spots may provide a potential matrix for assessing exposure to certain PFCs. We believe that blood spots, because of ease of collection compared with venipuncture, may play a role in the future for estimation of internal exposure to PFCs. To the best of our knowledge, partitioning ratios between blood spot and serum PFC levels have not yet been determined even as partial validation for the use of blood spots from whole blood instead of serum PFC measurements, the current gold standard for biomonitoring. We include previous dried blood spot results only as illustrative of other PFC exposure studies in children using different approaches rather than the validated (serum) methods we employed. Our study of Dallas children is representative only of children < 12 years of age who had blood samples drawn at Children’s Medical Center. Further research should be performed in a large representative sample to determine the serum concentrations of young children in the United States.

## Conclusions

In a convenience sample of 300 individual serum samples from a Dallas, Texas, medical facility, we detected four PFCs in at least 92% of the tested serum samples. Serum concentrations were lowest in children age 0 to < 3 years, and concentrations of some PFCs increased from birth through < 13 years of age. Major changes in PFC manufacturing, including discontinued production of PFOS and of PFOA at some U.S. facilities, occurred years before sample collection for this study. Our results show that several PFCs were detectable in children in Dallas, Texas, in 2009, including those born after changes in PFC manufacturing occurred. Data from a larger, more representative sample are needed to determine current PFC concentrations in the general population of U.S. children and evaluate relevant pathways of exposure. Additionally, further epidemiological studies will be necessary to determine whether PFC exposures in children at the concentrations detected are associated with adverse health outcomes.
